# A user-friendly automatic toolbox for hand kinematic analysis, clinical assessment and postural synergies extraction

**DOI:** 10.3389/fbioe.2022.1010073

**Published:** 2022-11-10

**Authors:** Martina Lapresa, Loredana Zollo, Francesca Cordella

**Affiliations:** Department of Engineering, Research Unit of Advanced Robotics and Human-Centred Technologies, Università Campus Bio-Medico di Roma, Roma, Italy

**Keywords:** hand kinematic analysis, clinical assessment, grasp and movement quality, postural synergies, motion capture system, MATLAB toolbox

## Abstract

The clinical assessment of the human hand is typically conducted through questionnaires or tests that include objective (e.g., time) and subjective (e.g., grasp quality) outcome measures. However, there are other important indicators that should be considered to quantify grasp and movement quality in addition to the time needed by a subject to execute a task, and this is essential for human and artificial hands that attempt to replicate the human hand properties. The correct estimation of hand kinematics is fundamental for computing these indicators with high fidelity, and a technical background is typically required to perform this analysis. In addition, to understand human motor control strategies as well as to replicate them on artificial devices, postural synergies were widely explored in recent years. Synergies should be analyzed not only to investigate possible modifications due to musculoskeletal and/or neuromuscular disorders, but also to test biomimetic hands. The aim of this work is to present an open source toolbox to perform all-in-one kinematic analysis and clinical assessment of the hand, as well as to perform postural synergies extraction. In the example provided in this work, the tool takes as input the position of 28 retroreflective markers with a diameter of 6 mm, positioned on specific anatomical landmarks of the hand and recorded with an optoelectronic motion capture system, and automatically performs 1) hand kinematic analysis (i.e., computation of 23 joint angles); 2) clinical assessment, by computing indicators that allow quantifying movement efficiency (Peak Grip Aperture), smoothness (Normalized Dimensionless Jerk Grasp Aperture) and speed (Peak Velocity of Grasp Aperture), planning capabilities (Time to Peak Grip Aperture), spatial posture (Wrist and Finger Joint Angles) and grasp stability (Posture of Hand Finger Joints), and 3) postural synergies extraction and analysis through the Pareto, Scree and Loadings plots. Two examples are described to demonstrate the applicability of the toolbox: the first one aiming at performing a clinical assessment of a volunteer and the second one aiming at extracting and analyzing the volunteer’s postural synergies. The tool allows calculating joint angles with high accuracy (reconstruction errors below 4 mm and 3.2 mm for the fingers and wrist respectively) and automatically performing clinical assessment and postural synergies extraction. Results can be visually inspected, and data can be saved for any desired post processing analysis. Custom-made protocols to extract joint angles, based on different markersets, could be also integrated in the toolbox. The tool can be easily exploitable in clinical contexts, as it does not require any particular technical knowledge to be used, as confirmed by the usability evaluation conducted (perceived usability = 94.2 ± 5.4). In addition, it can be integrated with the SynGrasp toolbox to perform grasp analysis of underactuated virtual hands based on postural synergies.

## 1 Introduction

The kinematic analysis of the human hand is fundamental both from a clinical and scientific point of view. For what concerns clinical assessment, correct estimation of hand kinematics is needed to evaluate functional alterations of the hand as a result of traumatic or neurological events and to evaluate the performance of the hand in grasping and manipulation tasks (Light et al., 1999; Schwarz et al., 2019). At the same time, kinematic analysis is important to reproduce as faithfully as possible the kinematic structure of the hand on artificial devices such as prostheses and orthoses.

The typical hand clinical assessment procedures include the measurement of the range of motion, preferred by the medical personnel, and the functional assessment through the execution of Activities of Daily Living (ADLs), which is generally performed by therapists. The activities are in general reproduced while sitting in front of a table, in order to isolate the hand district and avoid compensatory movements with the upper body. In addition, the set of selected activities should include the main grasp types (i.e., tip, lateral, tripod, flexion, power and extension grasp) to test the functionality of the hand in the main prehensile patterns (Light et al., 1999).

The tests proposed in the last decades mainly evaluate the hand function by means of a combination of objective and subjective measurements (Light et al., 1999; Metcalf et al., 2007). The time to complete a task and the quantity of tasks completed in a preset time period were proposed as objective outcome measures, together with qualitative scores on the way the object was grasped as well as subjective ratings assigned by the therapist or the patient.

For example, the Southampton Hand Assessment Procedure (SHAP) (Light et al., 2002) was proposed as a tool for a standardized clinical assessment of pathological and prosthetic hands function. It computes the Index of Functionality, based on the time needed to complete a task, to evaluate the level of function attributable to specific prehensile patterns. In fact, subjects who cannot perform the natural grip for a specific object are expected to take longer by implementing an abnormal grip pattern to move the object.

The existing clinical tests typically provide objective outcome measures based on time. However, the time needed to complete a task is only one of the possible outcome measures that could provide information about the hand functionality. Therefore, in literature several other outcome measures, based on parameters such as speed, distance, posture and time, were proposed to quantify the hand functionality in healthy and pathological subjects by means of specific indicators. The systematic review by Schwarz et al. (2019) resumes the most important indicators that have been used in clinical applications and that allow retrieving information about hand movement efficiency, planning, smoothness, spatial posture and speed, to quantify grasp and movement quality. All these indicators allow objectively assessing hand functionality because they are based on parameters measured by means of high-accuracy systems such as optoelectronic motion capture systems. However, calculating those indicators is not trivial because they entail a complete kinematic analysis of the hand and the extraction of distances between specific hand points, velocities, joint angles.

In literature, a lack of tools or applications that allow the user to automatically compute all these indicators is evident. In fact, all the studies found in literature, that provided a clinical assessment based on the previously listed parameters, required those indicators to be computed case by case by trained staff.

Some tools were also introduced in the last years to perform simulation and analysis of human or robotic grasp. For example, GraspIt! (Miller and Allen, 2004)! and Opengrasp (León et al., 2010) allow simulating grasps and evaluating them by means of numeric quality measures; and SynGrasp (Malvezzi et al., 2015) is a MATLAB (The Mathworks) toolbox that allows investigating the main grasp properties of fully or underactuated robotic and human hands. However, none of the previously mentioned tools is devoted to perform a complete clinical assessment of the hand functionality.

Given the extremely complex kinematic structure of the human hand, many studies have investigated the ways our central nervous system is able to control this very high number of Degrees of Freedom (DoFs) to perform dexterous movements. The concept of postural (or kinematic) synergies was first introduced by Santello et al. (1998). He demonstrated that the control of the hand posture in executing grasping tasks involves a few postural synergies that regulate the shape of the hand. Postural synergies are linear combinations of joint angles and can be extracted by applying dimensionality reduction techniques such as Principal Component Analysis (PCA) (Jolliffe, 2002). In particular, PCA allows reducing a large set of correlated hand motor variables into a smaller set of uncorrelated Principal Components (PCs), that are postural synergies. Many studies demonstrated that few PCs are able to explain most of the variance of the original data. In (Jarque-Bou et al., 2019) an in-depth analysis of the latest works about postural synergies extraction is provided.

Postural synergies were widely investigated not only to understand human motor control strategies (Jarque-Bou et al., 2019), but also in order to attempt to replicate these strategies on artificial devices (Catalano et al., 2014; Laffranchi et al., 2020), to build biomimetic robotic hands and to simplify the mechanical structure of hand prostheses through underactuation and develop advanced control strategies, to actuate a high number of DoFs with a compromise on weight and size of the devices. Therefore, the extraction of postural synergies is not only fundamental to understand individual motion strategies and their possible modifications due to musculoskeletal and/or neuromuscular disorders, but also to test biomimetic robotic hands.

To conclude, the human hand is an extremely sophisticated district both in terms of its kinematic structure and underlying control strategies. Therefore, assessing its functionality by means of quantitative indicators that take into account multiple aspects of movement and grasp quality results of primary importance to evaluate possible functional alterations. Moreover, the performance of bio-inspired artificial hands, developed to mimic the natural hand kinematics and dexterous capabilities (Romeo et al. (2021)), should be also assessed by means of the computation of quantitative indicators. The development of tools that allow automatically calculating all these objective outcome measures, could allow for an objective evaluation of hand functionality that could be conducted also by users with a limited technical knowledge. In addition, the automatic computation of kinematic synergies, which is likewise non trivial for non-technical users, could allow analyzing movement strategies of healthy and pathological subjects, as well as testing biomimetic capabilities of newly developed robotic hands and prostheses.

This paper proposes an open source toolbox for hand kinematic analysis, to be exploited in clinical contexts to evaluate some performance indicators of the hand in grasping tasks. The same tool can be also used to evaluate robotic and prosthetic hands. It can be exploited to quantify movement quality during the state-of-the-art performance tests (Jebsen et al., 1969; Thrope et al., 1989; Light et al., 2002; Jongbloed-Pereboom et al., 2013) as well as in custom-designed trials.

The tool proposed in this work represents a great advance with respect to the literature because it allows automatically calculating all the previously listed outcome measures, so that an objective evaluation of hand functionality could be conducted also by users with a limited technical knowledge. In addition, the tool provides the possibility of extracting and analyzing postural synergies by means of meaningful output plots and a customized virtual hand model which moves according to the synergy. To the best of our knowledge the existing tools that allow simulating and analyzing grasps generated by activating postural synergies (e.g. SynGrasp Malvezzi et al. (2015)), do not provide the possibility of extracting those synergies. Indeed, the user should perform this analysis by him/herself before exploiting SynGrasp to simulate grasps according to the selected synergies. With the proposed toolbox, instead, the extraction and analysis of postural synergies is integrated and there is the possibility of importing in SynGrasp the synergies as well as a custom virtual hand model created on the basis of the subject’s anthropometry. In this way, the proposed tool and SynGrasp become an integrated environment to extract synergies and simulate grasps with virtual hands.

This tool takes inspiration from the gait analysis tools integrated in several motion capture system softwares that are widely used by the clinical personnel. It aims at providing the possibility of completely inspecting hand functionality with the advantage of being compatible with any motion capture system, as it only requires loading marker positions data.

To sum up, with respect to other existing tools (Miller and Allen, 2004; León et al., 2010; Malvezzi et al., 2015) for grasping evaluation that only focused on simulation and analysis of human or robotic grasp, the proposed tool allows all-in-one analysis of: 1) hand kinematics, by reconstructing joint angles from the positions of markers placed on the hand; 2) performance indicators, that allow quantitatively assessing movement quality; 3) postural synergies, by extracting and interpreting individual motion strategies. Moreover, the tool can be integrated with the SynGrasp toolbox (Malvezzi et al., 2015) functionalities by incorporating the definition of a virtual hand model customized on the patient anatomy to perform grasp analysis of a custom hand controlled with the computed postural synergies. The developed licensed toolbox will be released on GitHub after the publication of the paper.

## 2 Materials and methods

### 2.1 Overview of the toolbox

**FIGURE 1:**
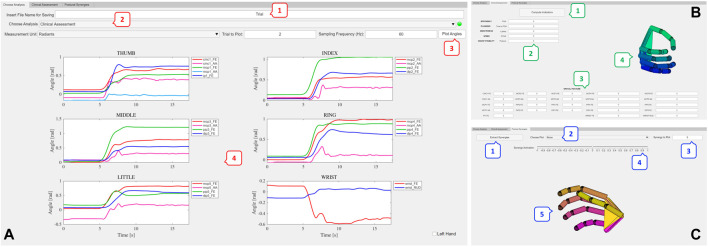
Overview of the toolbox. **(A)** Initial tab of the tool, which allows inserting the name of the MATLAB struct that will be saved (1). From the *Choose Analysis* dropdown menu (2) it is possible to choose the analysis to perform between *Clinical Assessment* and *Postural Synergies* extraction. Joint angles are automatically computed and the user can select the unit of measurement to visualize the joint angles, choose the trial to plot and insert the sampling frequency of data. Then, by pressing the *Plot Angles* button (3), angles can be inspected (4). **(B)** The *Clinical Assessment* tab allows computing the performance indicators that quantify movement quality (1,2) and the spatial posture of the hand (3), i.e., the range of motion. The customized virtual hand model reproducing the trial static hand posture can be visualized and saved (4). **(C)** The *Postural Synergies* tab allows automatically extracting postural synergies by pressing the *Extract Synergies* button (1). Meaningful plots (Pareto, Scree Plot, Loadings Plot) can be selected and inspected by the user (2). The synergy to be analyzed can be chosen by inserting the synergy number in the *Synergy to Plot* box (3). The slider (4) allows moving the customized virtual hand (5) according to the selected synergy.

This paragraph presents an overview of the toolbox for kinematic analysis and postural synergies extraction, shown in Figure 1.

The different types of analyses that can be conducted are organized in different tabs. In the upper part of the initial tab of the tool (Figure 1A) there is a field to insert the file name of the MATLAB struct that will be saved with all the extracted data.

The “*Choose Analysis*” tab allows choosing the type of analysis to perform between “*Clinical Assessment*” and “*Postural Synergies.*” The acquisitions that need to be recorded with the motion capture system and loaded in the tool are different according to the type of analysis that needs to be conducted, as it will be in depth described in Section 2.2.

Once the type of analysis is chosen, the tool automatically computes joint angles. Angles are saved in radiants into a MATLAB struct in the current folder and the struct is organized in order to have different fields ordered by the trial number of the loaded files.

As shown in Figure 1, angles can be visualized both in degrees and radiants and the possibility of selecting the trial to inspect is provided.

The “*Clinical Assessment*” tab (Figure 1B) allows computing the performance indicators to quantify movement quality, and visualizing meaningful plots and the hand posture for the loaded trial. Performance indicators as well as the customized virtual hand can be saved into the MATLAB struct.

Finally, the “*Postural Synergies*” tab (Figure 1C) allows extracting and analyzing subject-specific postural synergies, by inspecting plots such as the pareto plot, the scree plot and the loadings plot. The movement of the virtual customized hand according to a selected synergy can be visualized taking advantage of some functions implemented in SynGrasp. Results from postural synergies extraction can also be saved into the MATLAB struct.

The MATLAB toolbox will be released in the *mlapp* format and the Apache license, version 2.0, will be applied to the software. The toolbox code will be visible and modifiable by the users, except for the script which computes joint angles. The user could use the proprietary protocol already implemented in the tool or develop a custom protocol to extract joint angles and easily integrate it in the code to perform all the analyses. Some guidelines will be also provided in the code and in a *readme.txt* file to develop the joint angles computation script, to inform the user about the essential steps that should be included in the script (i.e., it should take as input the marker trajectories, compute and post-process the angles and save them into a MATLAB struct with a specific format).

### 2.2 Input and output data

The proposed toolbox, developed in MATLAB R2021b, takes as input the position of retroreflective markers placed on specific anatomical landmarks of the hand. Marker positions are recorded using an optoelectronic motion capture system. In particular, the BTS Smart-D optoelectronic system was used to record marker positions. It includes 8 infrared cameras with a resolution up to 1.4 Mp, acquisition frequency 60 Hz and accuracy ≤0.2 mm in a volume of 3 × 2 × 2 m^3^.

The positions of the markers placed on the hand, for a given trial, should be saved into a text file organized to have the *x*, *y* and *z* coordinates of each marker, with respect to the global reference frame of the motion capture system, in different columns. For example, given a marker *M*, data must be organized to have *M*_*x*_, *M*_*y*_ and *M*_*z*_ coordinates in three separate columns.

The files stored in the user PC that are going to be loaded in the toolbox should be located in a folder and named with progressive numbers (e.g., *Trial*_1_, *Trial*_2_, …). In the case of the clinical assessment procedure, two trials should be recorded and uploaded in the tool: the calibration trial which is needed to compute the *Posture* indicator (see Section 2.3.2), and the reach-to-grasp trial on which to perform the clinical assessment. The tool enables the user to select the file which corresponds to the trial for the assessment and the calibration file containing the full angular excursions recorded in a specific trial. Then, the trial to be inspected can be selected by inserting the number of the trial to be plotted in the “Trial to Plot” field. The “*Clinical Assessment*” tab allows computing the performance indicators to quantify movement quality. All the indicators related to a trial will be saved in the MATLAB struct under the field “*Trial*_*i*_”, where *i* is the trial number. In addition, the tool allows automatically saving to the MATLAB struct, for each analyzed trial, the computed joint angles in radiants, the marker positions and the customized virtual hand model created on the basis of the patient anatomy. In the case of kinematic synergies extraction, the tool enables to select the folder in the user PC with the trials recorded to perform postural synergies extraction (e.g., reach-to-grasp tasks of different objects), numbered with progressive numbers. All the trials will be automatically loaded after selecting “*Postural Synergies*” from the “*Choose Analysis*” tab and joint angles will be stored in the MATLAB struct, together with marker positions, extracted synergies (i.e., eigenvectors, eigenvalues, variance, principal component scores) and the customized virtual hand. The trial number for which the user wants to inspect joint angles can be inserted in the “*Trial to Plot*” field.

The methods to compute the joint angles, the indicators for hand clinical assessment and to extract postural synergies, and the performance metrics used to evaluate the usability of the toolbox will be described in the next Section.

### 2.3 Computational methods and algorithms

#### 2.3.1 Joint angles computation

In the example provided in this paper, a configuration of 28 hemispheric markers with a diameter of 6 mm, based on the protocol proposed in (Cordella et al., 2014), is used to extract 23 hand joint angles. However, the user is given the possibility of developing custom-made protocols to extract joint angles based on different markersets.

**FIGURE 2:**
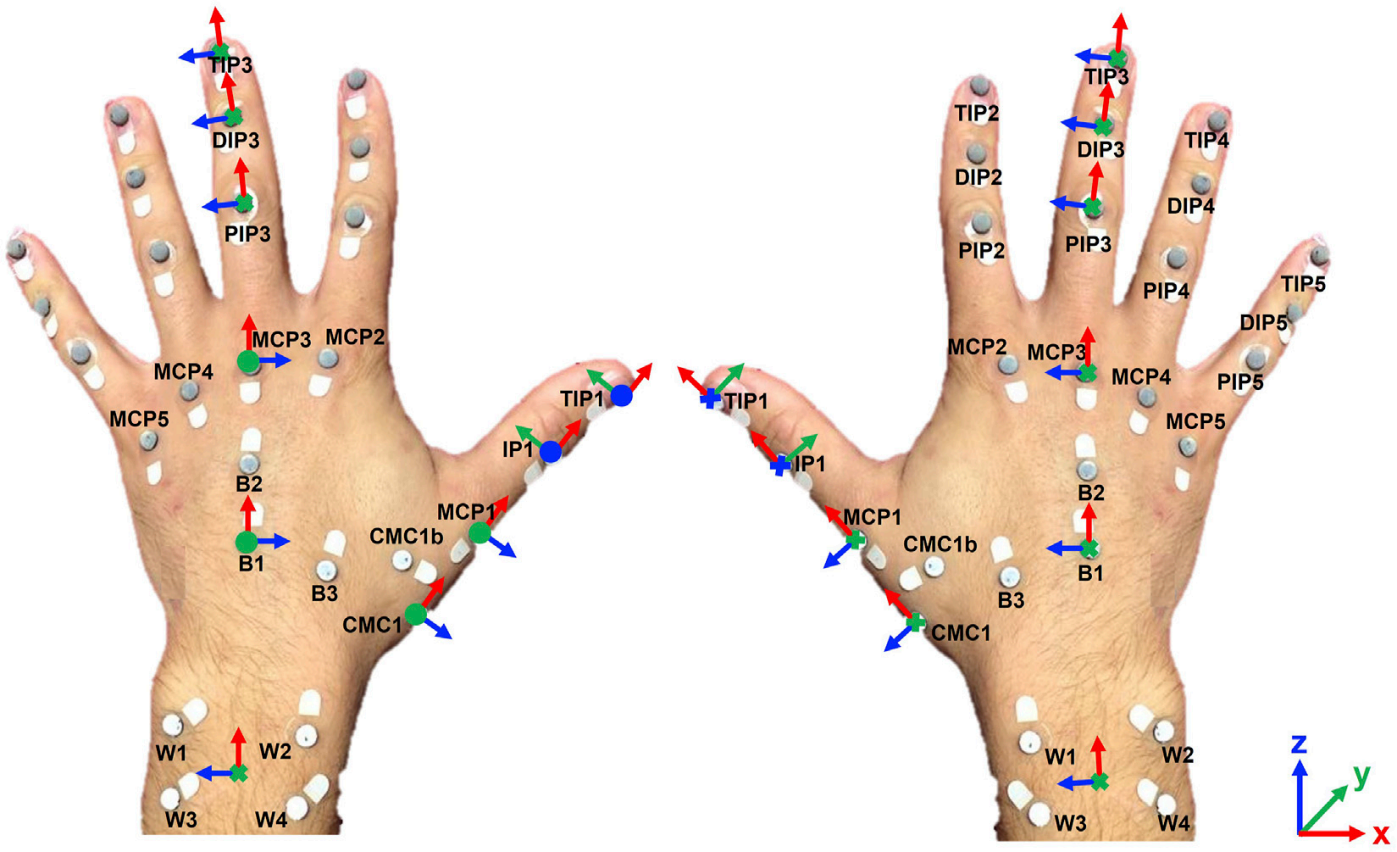
Markers placement on specific anatomical landmarks and local joint reference frames definition for the right and left hand.

According to the protocol used in this work, markers must be positioned on the carpometacarpal (CMC) joint of the thumb, metacarpophalangeal (MCP) joints of the five fingers, interphalangeal (IP) joint of the thumb, proximal interphalangeal (PIP) and distal interphalangeal (DIP) joints of the long fingers, and wrist. An additional marker (CMC1b) is placed to ameliorate the estimation of the adduction/abduction (AA) axis of the CMC joint. To properly extract joint angles with the proposed tool, markers must be named according to nomenclature shown in Figure 2.

Markers B1, B2 and B3 are positioned on the hand dorsum to define the hand local reference frame (i.e., hand base), with the *x* axis defined as the direction pointing from B1 to B2, the *y* axis defined as the cross product between the unit vectors $\vec{B1B3}$ and $\vec{B1B2}$ and the *z* axis which completes the frame. Markers W1, W2, W3 and W4 are placed on the wrist to define the wrist local reference frame, with the *x* axis defined as the direction which points from W3 to W1, the *y* axis as the unit vector normal to the plane defined by W1, W2, W3 and W4 and concordant to *y* axis of the hand base reference frame, and *z* axis which completes the frame.

The kinematic protocol implemented in this tool, proposed in (Cordella et al., 2014), allows reconstructing 21 hand joint angles by computing the relative rotation of local reference frames defined at the joints (see Figure 2). In particular, it allows calculating the AA angles of the CMC and MCP joints and the flexion/extension (FE) angles of the CMC, MCP, IP, PIP and DIP joints.

In addition, the protocol was completed with the estimation of the wrist FE and radio/ulnar deviation (RUD) angle. The relative rotation of the hand base local reference frame with respect to the wrist local reference frame is computed as a sequence of Euler angles (ZYX) so that the FE is the rotation of the hand base reference frame around the *z* axis of the wrist frame, and the RUD angle is the rotation of the hand frame around the *y* axis of the wrist frame. In particular, the accuracy of the wrist angles estimation was demonstrated by computing the reconstruction error between the real B1 marker 3D coordinates with respect to the hand base reference frame given by the motion capture system, and the reconstructed B1 3D coordinates with respect to the same reference frame, computed starting from the extracted wrist angles. Errors were computed for 21 reach-to-grasp trials (i.e. the trials exploited to extract postural synergies in Section 3.2). On average, errors were in the order of 3.24 ± 1.85 mm, 1.53 ± 0.44 mm and 0.60 ± 0.41 mm in the *x*, *y*, *z* directions respectively.

The convention of the angle signs is reported in Table 1.

**TABLE 1:** Angles signs.

Angle	**+**	**−**
AA Fingers	Toward the thumb	Toward the little
FE Fingers	Flexion	Extension
FE Wrist	Flexion	Extension
RUD Wrist	Radial Deviation	Ulnar Deviation

Before computing joint angles, potential missing values in marker positions data due to marker occlusions are identified and replaced with the nearest non-missing value. However, this elaboration step follows preliminar precautions that should be considered when performing an experimental acquisition with an optoelectronic motion capture system. In particular, the experimental setup should minimize the risk of marker occlusions by properly positioning the cameras, and a first step of labelling and gap filling should be executed with the proprietary software. Then, joint angles are computed with the kinematic protocol and filtered first with a Hampel filter with a 30-samples window to remove potential outliers and second with a moving mean filter on a 30-samples window to smooth data.

The kinematic protocol can be used to analyze the right and the left hand. In particular, if the user wants to analyze the left hand, the checkbox “Left Hand” must be marked in the initial tab of the toolbox. The protocol will be automatically adapted for the left hand by switching the sign of the FE and AA angles of the CMC1, MCP1, MCP joints of the long fingers and the sign of the RUD angle of the wrist. The rest of the analyses (i.e. clinical assessment and postural synergies extraction) is independent from the analyzed hand.

#### 2.3.2 Indicators for automatic hand kinematic assessment

The “*Clinical Assessmen*t” tab of the toolbox allows calculating important performance indicators that can be exploited to evaluate the hand functionality. The selected indicators allow retrieving information about movement efficiency, planning, smoothness, speed and spatial posture and were previously exploited in literature to quantify movement quality in clinical contexts, taking advantage of motion capture systems to perform kinematic analysis (Schwarz et al., 2019). In particular, the following indicators have been implemented in this toolbox:

• *Peak Grip Aperture* (*PGA*): it provides information about the efficiency of the hand in executing a grasping task. It is computed as the maximal value of the grip aperture during a reach-to-grasp movement, that is the maximum distance between markers TIP1 and TIP2 over time:

$$PGA=max\left(dist_{\mathrm{TIP1-TIP2}}\left(t\right)\right)\;\left[mm\right]$$ (1)

where *t* is the time step. The *PGA* was used in (Patterson et al., 2011) to evaluate movement efficiency in 18 post-stroke patients and 9 healthy subjects.

• *Time to Peak Grip Aperture* (*t*_*PGA*_): it provides information about the planning capabilities of the subject. It is calculated as the time from movement onset until peak grip aperture relative to the duration of the movement. Movement onset is computed as the time when the norm of the movement velocity, defined as the first derivative of B1 trajectory, is about 10% of the peak velocity:

$$NormVelocity=\|\frac{dB\vec{1}\left(t\right)}{dt}\|\;\left[mm/s\right]$$ (2)

$$PeakVelocity=max\left(NormVelocity\right)\;\left[mm/s\right]$$ (3)

$$t_{\mathrm{Onset}}=t_{10\%PeakVelocity}\;\left[s\right]$$ (4)

therefore

$$t_{\mathrm{PGA}}=\left(\frac{t_{\mathrm{PeakGripAperture}}-t_{\mathrm{Onset}}}{t_{\mathrm{End}}-t_{\mathrm{Onset}}}*100\right)\;\left[\%\right]$$ (5)

where *t*_*End*_ is the time when *NormVelocity* is 10% of the peak velocity after the peak occurred (Lauretti et al. (2017)). The *t*_*PGA*_ was exploited by Baak et al. (2015) to investigate deficits of reach-to-grasp coordination after stroke in 16 post-stroke and 16 healthy control subjects.

• *Normalized Dimensionless Jerk Grasp Aperture* (*J*_*Grasp*_): it provides information about the smoothness of the grasp action. It was introduced in (van Kordelaar et al., 2014) to assess the quality of motor control of the paretic upper limb after stroke. It is computed as:

$$J_{\mathrm{grasp}}=\sqrt{\frac{1}{2}*\int_{t_{\mathrm{Onset}}}^{t_{\mathrm{End}}}j{\left(t\right)}^{2}\;dt*\frac{MD^{5}}{L_{\mathrm{grasp}}^{2}}}$$ (6)

where *j*(*t*) is the jerk of the grasp aperture (i.e. the third time derivative of the grasp aperture), *MD* is the movement duration computed as *MD* = *t*_*End*_ − *t*_*Onset*_ and *L*_*grasp*_ is the difference in grasp aperture between the start and the end of the reach-to-grasp movement.

• *Peak Velocity of Grasp Aperture* (*PVGA*): it is the maximal value of the aperture rate, computed as:

$$PVGA=max\left(\frac{d\left(dist_{\mathrm{TIP1-TIP2}}\left(t\right)\right)}{dt}\right)\;\left[mm/s\right]$$ (7)

It provides information about the speed of the movement. Lang et al. (Lang et al., 2005) used this indicator to quantify the movement speed in patients with acute hemiparesis and healthy subjects performing reach and reach-to grasp tasks.

• *Posture of Hand Finger Joints*: this indicator allows evaluating grasp stability given the hand joints posture during the static grasp phase (León et al., 2012). It measures how far each joint is from its maximum and minimum range of motion and it can be computed as

$$Posture=\frac{1}{N}\overset{N}{\underset{i=1}{\sum }}{\left\{\frac{y_{i}-a_{i}}{R_{i}}\right\}}^{2}$$ (8)

where *N* is the number of joints, *a*_*i*_ is the middle range position defined as the relaxed hand posture and *R*_*i*_ is the joint angle range between *a*_*i*_ and either the upper or lower angle limit used to normalize the index. In particular, *R*_*i*_ is defined as

$$R_{i}=\left\{\begin{array}{c} a_{i}-y_{im}\text{ if }y_{i}<a_{i}\;\\ y_{iM}-a_{i}\text{ if }y_{i}>a_{i}\; \end{array}\right.$$ (9)

where *y*_*iM*_ and *y*_*im*_ are the maximum and minimum angle limits of the joint *i*. To have 1 as its best value, the index is modified as *Posture* = 1 − *Posture*.

• *Wrist FE Angle*: it is the range of the wrist FE angle during the reach to grasp movement.
• *Wrist AA Angle*: it is the range of the wrist AA angle during the reach to grasp movement.
• *Finger FE Angle*: it is the range of the fingers FE angle during the reach to grasp movement.

*Wrist FE Angle*, *Wrist AA Angle* and *Finger FE Angle* regard the spatial posture of the hand and were exploited in (Michaelsen et al., 2004) to evaluate compensation strategies in grasping tasks in adults with hemiparesis and in (Beebe and Lang, 2009) as metrics to predict upper extremity function after stroke.

**FIGURE 3:**
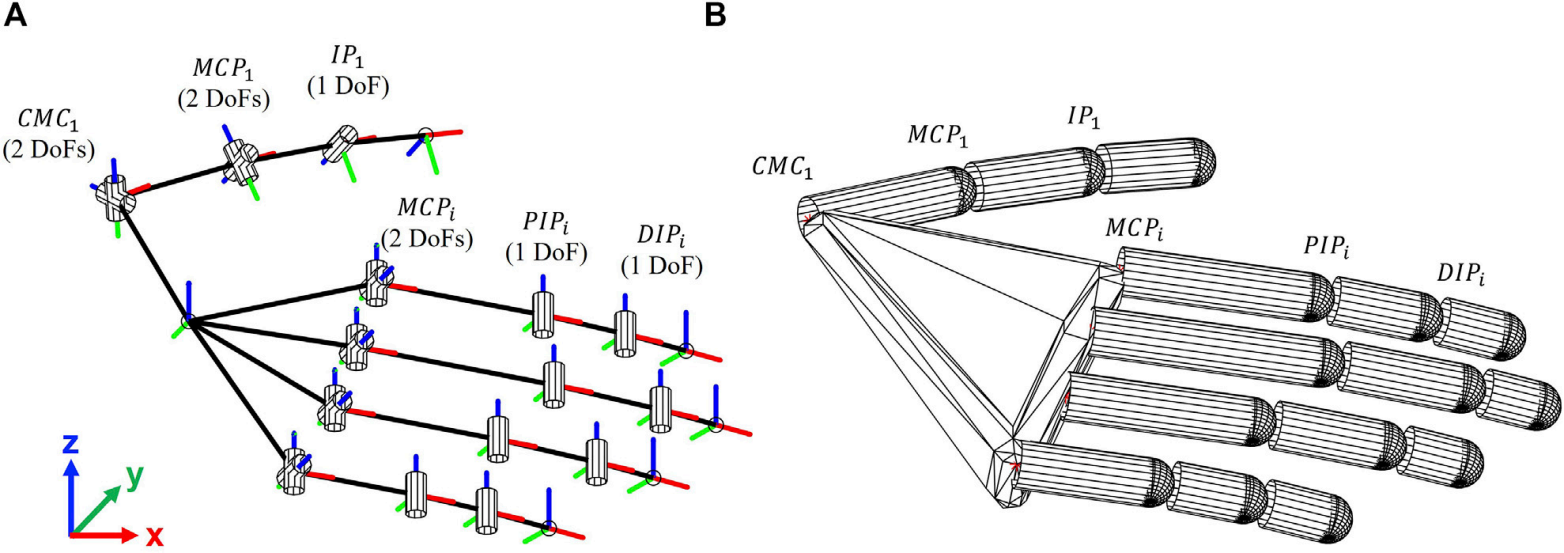
**(A)** 21-DoFs hand model with joints, axes and DoFs highlighted; **(B)** 21-DoFs customized virtual hand developed in SynGrasp. *i* = 2 : 5 stands for index, middle, ring and little finger respectively.

In addition to the computation of meaningful performance indicators, the tool allows visualizing the hand posture of the subject during the grasp trial by means of a custom virtual hand developed taking advantage of the functions implemented in the SynGrasp toolbox (Malvezzi et al., 2015). The basic hand models integrated in SynGrasp consist of hands with fixed dimensions and with fixed frames orientations. For example, the paradigmatic hand in SynGrasp is a 20 DoFs hand with pre-defined phalanges lengths and frames orientations. In addition, the thumb only includes 4 DoFs (FE and AA of the MCP, and FE of the IP), even though the CMC1 joint is fundamental for properly modelling the thumb AA and FE. The custom virtual hand proposed in this work, instead, modifies the model of the thumb by including the two DoFs of the CMC1 joint. In addition, the lengths of the phalanges (i.e. the distance between adjacent reference frames) and the orientation of the reference frames are customized for the subject taking as input the position of markers placed on specific anatomical landmarks (i.e., before the joint of the hand to reduce soft-tissue artifacts). The lengths of the phalanges are calculated as the distance between two consecutive markers placed on the anatomical joints, whereas the orientation of the frames is calculated by taking as input the reference frames defined from the markers. Both parameters are related to an initial, relaxed posture of the hand, corresponding to the initial posture of the trials (i.e., hand on the table in relaxed position). Figure 3 reports the customized 21-DoFs hand with joints, axes and DoFs highlighted. This novel virtual hand leads to a more faithful representation of the real hand posture with respect to the basic hands provided in the SynGrasp toolbox.

#### 2.3.3 Postural synergies extraction and analysis

Another important functionality of the proposed toolbox is the postural synergies extraction and analysis. As previously defined in Section 1, postural synergies represent patterns of joint angles activation that regulate the shape of the hand in grasping tasks (Santello et al., 1998). By extracting and analyzing subject specific postural synergies, one can inspect individual motion strategies and use this information on the one hand to gather pathological changes due to, for example, neurological or musculoskeletal disorders, and on the other hand to attempt to reproduce those motion strategies with artificial systems, such as hand prostheses and exoskeletons.

Postural synergies can be extracted through PCA (Jolliffe, 2002), which is a technique that allows reducing the dimensionality of correlated variables in a data set by transforming them to a new set of uncorrelated variables, that is the PCs, ordered so that the first few PCs retain most of the variation present in the data set.

To extract postural synergies, multiple trials that contain marker positions recorded in static grasping tasks must be loaded in the toolbox.

The data matrix is organized to have as rows the observations (i.e., the static postures) and as columns the variables (i.e., the angles). In general, PCA can be applied to the covariance matrix or to the correlation matrix of data. In this work, PCA is applied on the correlation matrix, as each variable (i.e., joint angle) could be subject to different changes of scale (Jolliffe and Cadima, 2016). This algorithm computes the matrix of the coefficients (or loadings) *C* which contains as columns the eigenvectors of the correlation matrix ordered in descending importance, the eigenvalues vector *E*_*val*_, the principal component scores *S* (i.e., the data expressed in the new principal component space) and the explained variance by each principal component *E*.

The percentage of variance explained by each PC (or synergy) can be observed through a pareto plot, that reports the PCs and their variances up to the component that explains 95% of the original data variance.

The number of PCs to be considered can be chosen taking advantage of the scree plot (Cattell, 1966) provided in the tool, that reports the eigenvalues *E*_*val*_ ordered from the largest to the smallest versus the number of components. The optimal number of PCs corresponds to the point beyond which the scree plot defines a more or less straight line. In particular, the first point on the straight line is taken as the last component to be considered, and, if there are two or more straight lines formed by the lower eigenvalues, then the optimal PCs number is taken as the upper end of the left-most straight line. The knee point of the scree plot is automatically identified in the tool to define the optimal number of PCs.

Finally, the importance of the joint angle variables to each synergy can be investigated through a bar plot that reports the absolute values of the loadings of each PC and predefined thresholds. The thresholds allow systematically assign a certain level of importance to the joint angle. In particular, in this tool, the thresholds are set to 25%, 50% and 75% of the maximum absolute value of the loading for the considered synergy.

The movement resultant from the activation of each extracted synergy can be visualized through the 21-Dofs virtual hand, previously described. The customized hand can be saved in the MATLAB struct and it can be imported in SynGrasp and used as a custom-designed hand to investigate additional grasp properties such as controllable forces and object displacement, manipulability analysis, grasp quality measures. The extracted postural synergies can be saved in the MATLAB struct and eventually imported into the SynGrasp toolbox and used to define coupling strategies between joints to simulate an underactuated control, in order to perform grasp analysis using the novel customized virtual hand underactuated on the basis of the computed synergies. Therefore, the tool proposed in this work and SynGrasp become an integrated environment that allows extracting synergies and simulating grasps with customized virtual hands.

### 2.4 Usability evaluation

The usability of the tool was evaluated in terms of its effectiveness, efficiency and satisfaction, according to the definition of usability proposed in the ISO 9241-11: “the extent to which a product can be used by specified users to achieve specific goals with effectiveness, efficiency and satisfaction in a specified context of use” (Fernandez et al. (2011); Iso, (1998)).

In particular, the satisfaction of the user was evaluated through the Usability Metric for User Experience (UMUX) proposed in Finstad (2010). It is a four-item Likert scale that allows assessing the perceived usability of an application. The four items are listed in the following:

(1) The tool capabilities meet my requirements (1 strongly disagree - 7 strongly agree)
(2) Using the tool is a frustrating experience (1 strongly disagree - 7 strongly agree)
(3) The tool is easy to use (1 strongly disagree - 7 strongly agree)
(4) I have to spend too much time correcting things with the tool (1 strongly disagree - 7 strongly agree)

The final score of perceived usability for each subject is obtained by rescaling odd items scores as (*score* − 1) and even items scores as (7 − *score*). Then, to obtain the final score between 0 and 100, the following equation is applied:

$$Usability=\frac{\sum_{i=1}^{4}score_{i}}{24}*100.$$ (10)

In this work, eight volunteers were recruited to evaluate the usability of the tool. They were asked to use the tool to perform all the analyses. Then, the UMUX questionnaire was administered to evaluate the perceived usability. Scores from all the volunteers were then averaged to obtain an overall score regarding the usability of the tool.

In addition, we decided to evaluate the efficiency and effectiveness of the tool through specific metrics used in literature (Saleh et al. (2017)). For the efficiency, we measured several task times: 1) the time to load data and perform joint angle computation; 2) the time to compute the indicators for clinical assessment and to extract postural synergies and 3) the time to visualize the plots and the hand moving according to the selected synergy. The indicators related to times were computed for the specific hardware used in this application, i.e. a 64-bit HP Pavilion Laptop 15 with processor AMD Ryzen 7 5700U and 16 GB RAM. The effectiveness was evaluated through: 1) the number of navigational steps, by measuring the number of pages accessed to perform a specific type of analysis; 2) the number of errors the participants made when using the tool and 3) the number of mouse clicks for each tab (i.e. choose analysis, clinical assessment, postural synergies extraction).

## 3 Illustrative examples and results

### 3.1 Clinical assessment

A healthy young man aged 26, with hand length (HL) 18.5 cm measured from the tip of the middle finger to the distal wrist crease, was recruited to perform a grasping task of a cylindrical object with 5 cm of diameter, 10 cm of height and 500 g of weight. 28 hemispheric retroreflective markers with a diameter of 6 mm were placed on the volunteer hand according to the marker placement shown in Figure 2. Markers positions were recorded using the BTS Smart-D optoelectronic system.

The participant was asked to sit in front of a table in a comfortable position. Before performing the grasping task, a calibration trial was recorded, in which the volunteer performed maximal FE and AA of each finger joint. This trial is needed as the *Posture of Hand Finger Joints* indicator needs the maximum and minimum angle limits of each joint to be computed. Then, the volunteer performed the grasping task: starting with the dominant hand in neutral position on the table, the participant grasped the cylinder and kept the static grasp posture for 3 s.

Marker positions were recorded and processed with the BTS software to export a *.emt* file with marker coordinates ordered as previously explained.

The first step to be performed in the developed tool is to choose the file name to save data in the MATLAB struct. Then, the *.emt* files are imported by selecting “*Clinical Assessment*” in the “*Choose Analysis*” field and joint angles are automatically computed and saved, to make them available for any post-processing analysis.

The tool allows inspecting the computed angles by simply pressing the *“Plot Angles”* button on the main tab once the number of the trial to plot is inserted. The sampling frequency of the motion capture system must also be inserted to plot the angles. The measurement unit can be chosen between degrees and radiants. Figure 1A shows the angles computed for this representative trial. Each finger and wrist corresponds to a subplot.

Once joint angles are extracted, the “*Clinical Assessment*” tab allows calculating meaningful performance indicators, listed in Section 2.3.2. The results regarding the efficiency, planning capabilities, smoothness, speed and grasp stability are highlighted with different color shades in the tool, according to their computed values with respect to normative reference values. This is done to provide the clinicians with an immediate intuitive visual feedback. Tables 2, 3 report the computed indicators for this trial. In particular, in Table 3 the absolute range is computed as the difference between the maximum and minimum angle excursion, where flexion, abduction and radial deviation are positive, and extension, adduction and ulnar deviation are negative, as reported in Table 1. To be calculated, the *Posture* indicator needs the calibration trial that is loaded together with the grasp trial.

**TABLE 2:** Computed Indicators regarding efficiency, planning capabilities, smoothness, speed and grasp stability.

Indicator	Value
*PGA*	117.5 mm
*t*_*PGA*_	49%
*J*_*grasp*_	5.98*10^5^ [-]
*PVGA*	162.5 mm/s
*Posture*	0.82 [-]

**TABLE 3:** Computed Indicators regarding the angle range.

Indicator	Absolute range [rad]	Min [rad]	Max [rad]
*CMC*1_*FE*_	0.57	0.12	0.69
*CMC*1_*AA*_	0.53	−0.09	0.44
*MCP*1_*FE*_	0.51	0.03	0.54
*MCP*1_*AA*_	0.17	−0.16	0.01
*IP*1_*FE*_	0.72	0.07	0.80
*MCP*2_*FE*_	0.53	0.05	0.58
*MCP*2_*AA*_	0.36	0.01	0.38
*PIP*2_*FE*_	0.92	0.14	1.06
*DIP*2_*FE*_	0.65	0.04	0.69
*MCP*3_*FE*_	0.78	0.01	0.79
*MCP*3_*AA*_	0.35	0.04	0.40
*PIP*3_*FE*_	1.20	0.03	1.23
*DIP*3_*FE*_	0.48	0.08	0.56
*MCP*4_*FE*_	0.99	0.01	0.99
*MCP*4_*AA*_	0.39	−0.06	0.33
*PIP*4_*FE*_	0.80	0.09	0.89
*DIP*4_*FE*_	0.67	0.06	0.74
*MCP*5_*FE*_	0.78	0.05	0.83
*MCP*5_*AA*_	0.55	−0.33	0.22
*PIP*5_*FE*_	0.50	0.17	0.66
*DIP*5_*FE*_	0.59	0.08	0.67
*WRIST*_*FE*_	0.74	−0.61	0.13
*WRIST*_*RUD*_	0.19	−0.11	0.08

**FIGURE 4:**
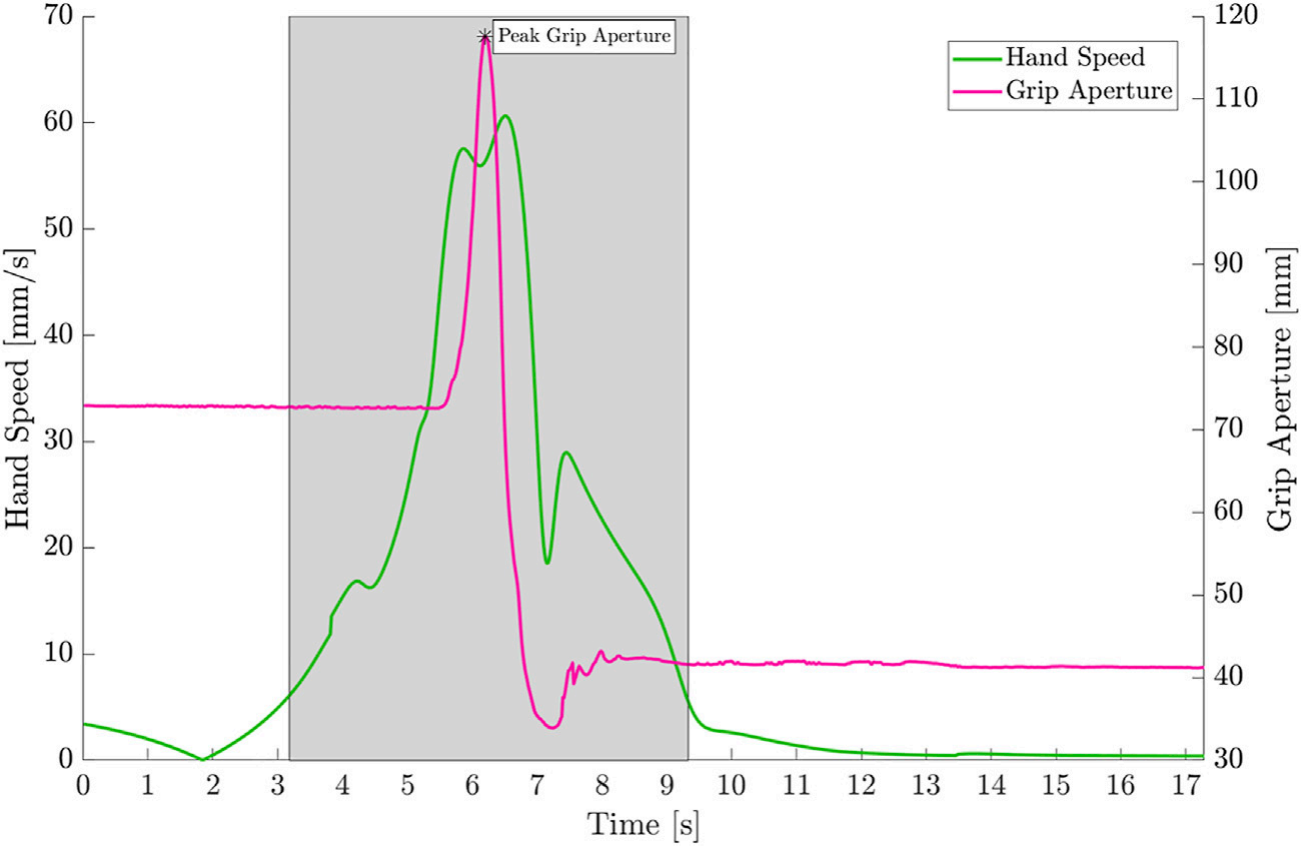
Hand Speed (green) and Grip Aperture (magenta) for one representative subject in the reach-to-grasp task of a cylindrical object. The area highlighted in light gray corresponds to the time between movement onset and movement end. The PGA is also reported in the plot.

Moreover, Figure 4 reports the hand speed and grip aperture for this representative trial. It is evident the movement onset and movement end, computed on the basis of the maximum hand speed, as explained in Section 2.3.2. The light gray shaded area corresponds to the reach-to-grasp phase of the movement. The *PGA* is also highlighted.

**FIGURE 5:**
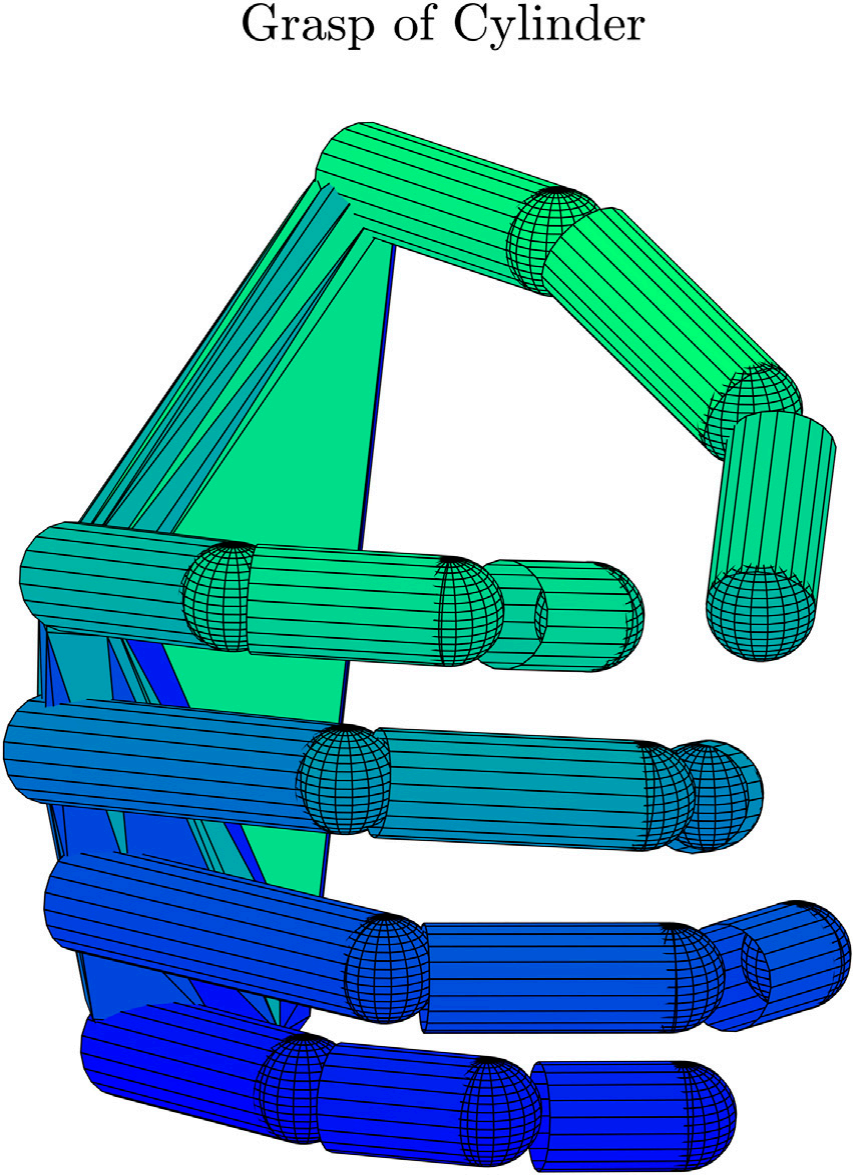
21 DoFs virtual hand that reproduces the static grasp.

Finally, the static posture executed by the subject can be visualized as shown in Figure 5 by exploiting the customized virtual hand model implemented in this tool.

### 3.2 Postural synergies

The volunteer enrolled for the clinical assessment procedure also performed the experimental trials for postural synergies extraction. The participant was asked to perform grasping tasks with his dominant, right, hand. In particular, 28 markers were placed on the hand of the volunteer according to the protocol described in Section 2 and the marker positions were recorded using the BTS Smart-D optoelectronic system.

The volunteer sat on a chair in front of a table in a comfortable position and was asked to execute 21 grasps of objects commonly used in ADLs. The grasps were selected to cover the entire grasp taxonomy proposed by Cutkosky (1989). The trial started with the dominant hand in neutral position on the table and the non-dominant hand grasping the object, then the subject started the reaching phase and the object was passed from the non-dominant to the dominant hand, to execute a 3-s static grasp with the markerized hand. The trial stopped at the end of the static grasp phase.

The marker positions saved into a *.emt* file were imported into the proposed MATLAB tool by selecting *“Postural Synergies”* in the *“Choose Analysis”* field in the main tab. The tool allowed selecting the folder with the files properly named (i.e. *Trial*_1_, …, *Trial*_21_) and joint angles could be automatically computed and saved for each trial. As shown for the representative trial presented in Section 3.1, angles can be inspected by selecting the measurement unit, the sampling frequency and the trial in the main tab. Then, postural synergies were extracted by pressing the “*Extract Synergies*” button in the “*Postural Synergies*” tab.

Meaningful plots can be visualized in the tool by selecting the type of information to be investigated.

**FIGURE 6:**
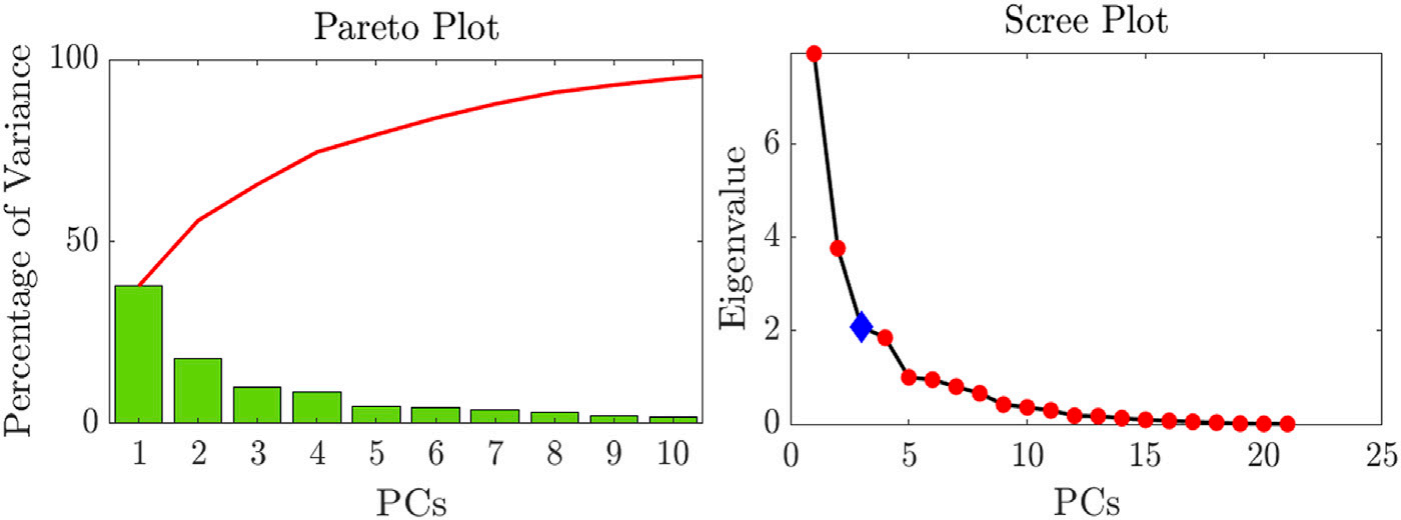
Pareto Plot showing the percentage of variance explained by each PC and Scree Plot used to choose the number of PCs to consider.

Postural synergies analysis revealed that this volunteer exploits three main synergies in executing the proposed grasping tasks. The Scree Plot shown in Figure 6 allows in fact to determine the number of PCs to consider. Results about the variance showed that with three PCs it was possible to explain more than 65% of the variance, and that with four PCs the 74% of the variance was explained. Figure 6 reports the percentage of variance explained by each PC up to the component that allows reaching 95% of the variance.

**FIGURE 7:**
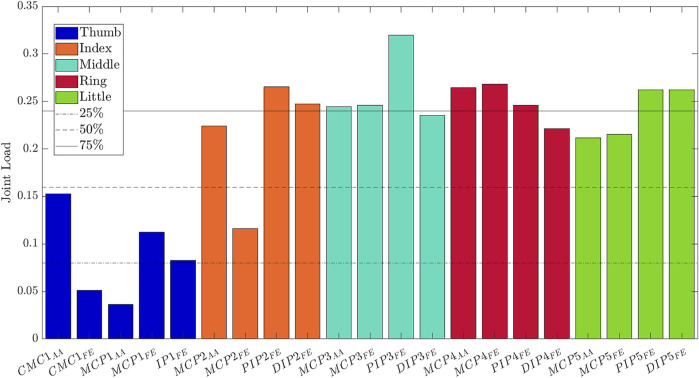
Loadings of the joint angles for the first PC, that is the first postural synergy.

The analysis of the loadings matrix *C* allows interpreting the extracted synergies in terms of the contribution of joint angles to the synergy. In particular, for the sake of brevity the first extracted synergy is reported in Figure 7. It is evident that this synergy is characterized by a major contribution of PIP and DIP joints excursion of all the long fingers, as well as a contribution of the MCP FE and AA movements. The thumb does not play an essential role in the first synergy, and the major contribution of this finger is given by CMC1 AA. On the other hand, in the second synergy the contribution of the thumb is more evident. The CMC1 FE and AA and the MCP1 AA give a major contribution to this synergy. Moreover, the second synergy also includes an important role of the MCP FE of the long fingers. Finally, the third synergy is characterized by the MCP1 and IP1 FE and by the MCP2 FE.

**FIGURE 8:**
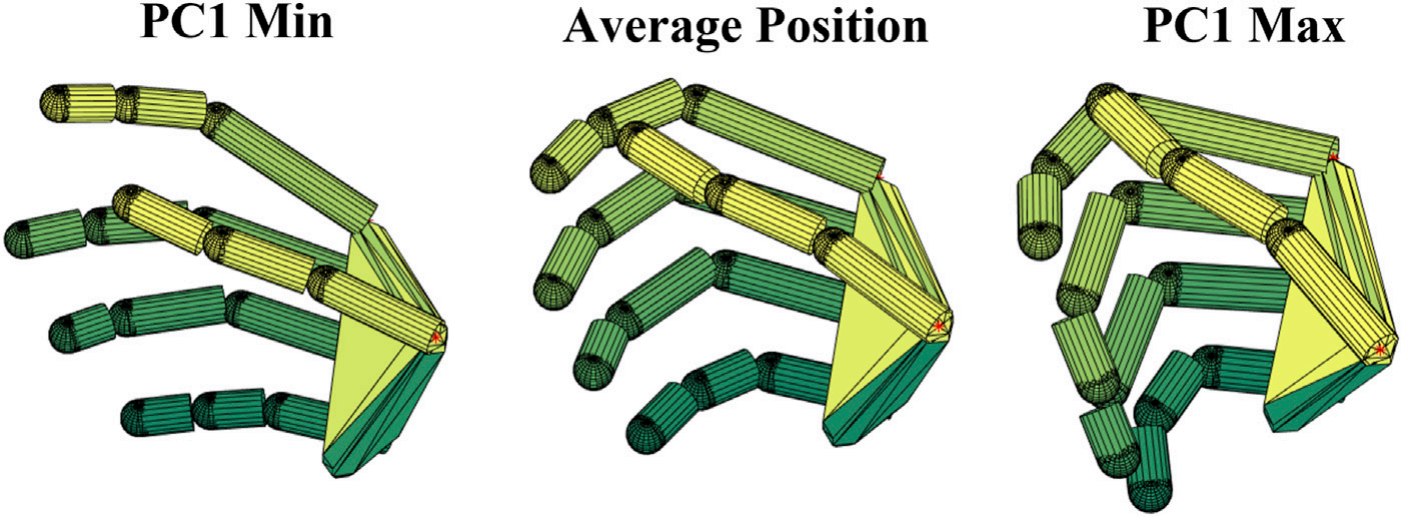
Movement of the hand according to the activation of the first postural synergy. PC1 Min corresponds to an extended hand, PC1 Max to progressive flexion of PIP, DIP and MCP joints.

The tool allows also visualizing the movement of the hand according to the activation of a postural synergy, through the 21 Dofs virtual hand purposely developed. Figure 8 shows the movement of the hand from extension (PC1 Min) to flexion (PC1 Max) according to the activation of the first synergy.

### 3.3 Usability results

Results about the usability of the tool are reported in Table 4.

**TABLE 4:** Evaluation of the usability of the toolbox.

	Item	Score
Satisfaction	Perceived usability	94.2 ± 5.4
Efficiency	Time to load data (1 trial)	30 s
	Time to load data (21 trials)	5 min
	Time to compute indicators (clinical assessment)	4 s
	Time to extract synergies (postural synergies)	3 s
	Time to visualize plots and hand posture	1 s
Effectiveness	Number of navigational steps	1 for each tab
	Number of errors	0
	Number of mouse clicks (choose analysis tab - clinical assessment)	8
	Number of mouse clicks (choose analysis tab - postural synergies)	7
	Number of mouse clicks (clinical assessment)	1
	Number of mouse clicks (postural synergies)	4

The perceived usability results represent the mean and standard deviation of the usability perceived by the eight volunteers. The times refer to the computational time of the tool. The time to load data is the most time-demanding part of the tool functionalities as it includes the loading, pre-processing of data and joint angle computation according to the protocol proposed in (Cordella et al., 2014). All the other functionalities require very little time. The time to compute the indicators for clinical assessment, which is about 4 s, includes the computation of the indicators, the preparation of figures and the visualization of the static grasp posture through customized virtual hand. Finally, results about the effectiveness items were the same for all the subjects. The error rate was assigned a score 0 since no errors are generated by the subjects in using the toolbox.

## 4 Discussion

The main novelty of the proposed open source toolbox consists in allowing to perform all-in-one kinematic reconstruction, clinical assessment and postural synergies extraction, according to the type of analysis to be performed.

The indicators are automatically computed by the tool, which is user-friendly and does not require a technical background to be used. In fact, the therapist/clinician only has to load data acquired with motion capture systems, which have became widespread in clinical applications and already used for other types of analyses such as gait analysis.

The motion capture acquisitions needed to perform these types of analyses can be selected according to the specific purpose: for the clinical assessment, a calibration trial in which the finger joints span the full range of motion needs to be recorded before performing the reach-to-grasp movements to be assessed. The grasps to be executed can be selected according to the state-of-the-art performance test to be conducted or chosen to perform a custom-designed trial. It is important to ensure that the principal prehensile patterns are executed to conduct the analysis, as supported by (Light et al., 1999). For the postural synergies extraction, multiple reach-to-grasp trials need to be executed. For example, in this paper 21 grasps were selected to be performed by the volunteer. These grasps also include prehensile patterns of the SHAP test, therefore the same acquisitions could be exploited both to conduct a clinical assessment and to extract postural synergies.

The tool enables to reconstruct joint angles from marker positions captured with any motion capture system. It is sufficient to save data from the motion capture system in text files with marker coordinates organized in columns. Moreover, the sampling frequency of the motion capture system used for the analysis can be manually inserted to allow visualizing angles and hand postures over time.

The protocol implemented in the tool allows reconstructing joint angles with high accuracy. In fact, errors between real and reconstructed marker positions were demonstrated to be in the order of 4 mm for what concerns the fingers (Cordella et al., 2014) and below 3.2 mm for what concerns the wrist. These latter errors could be related to soft tissue artifacts. In fact, during the grasping task marker B1 could slightly move in the *x* direction as the fingers flex. In addition, the marker is positioned near to the hand tendons which could generate surface movements of the markers. Despite the position of the markers was chosen to minimize soft tissue artifacts, errors related to skin movements are always present when using motion capture system. However, an error of about 3 mm in the *x* direction can be considered acceptable for the proposed application, as it is of an order of magnitude of about half the dimension of the used markers. Overall, these errors satisfy the accuracy expected for kinematic reconstruction purposes.

In this paper, two practical examples are also presented. The first example regards the clinical assessment of an healthy volunteer who performed a reach-to-grasp task of an object. Multiple indicators are computed to quantify movement quality. Results are in line with the performance of healthy controls already investigated in literature. In fact, typical healthy control values are in the range of 125 ± 18 mm for the *PGA*, 124 ± 26 mm/s for *PVGA*, and 41.6 ± 5.3% for *t*_*PGA*_, as reported in (Lang et al., 2005). Moreover, the movement time is shorter in healthy controls when compared to patients with neurological disorders (Lang et al., 2005). The *J*_*Grasp*_ indicator provides information about the smoothness of the reach-to-grasp movement. When assessing movement quality, the smaller the *J*_*Grasp*_ indicator, the better the performance (van Kordelaar et al., 2014). Finally, the *Posture* indicator provides information about the grasp stability and should have 1 as its best value. The healthy volunteer who performed the experiment reported 0.82 for the *Posture* indicator, which suggests good grasp stability. Indicators about the spatial posture, i.e. *Finger*_*FE*_, *Wrist*_*FE*_ and *Wrist*_*AA*_ were computed in the presented example and indicate the span of the considered joint angular range during the movement. However, these indicators could also be exploited to assess the range of motion of fingers and wrist in free movements. Assessment of range of motion of hand joints could be very useful to trace the effectiveness of the therapy in post-stroke patients, as the reduction of range of motion is one of the principal effects of stroke. In addition, more indicators could be implemented in this tool to enrich the clinical assessment.

The second example shows how to extract postural synergies from a healthy volunteer who performed 21 reach-to-grasp trials. For this volunteer, three main synergies were extracted. By activating the extracted synergies in combination, an artificial hand that is underactuated on the basis of these synergies should be able to replicate with high fidelity most of the original hand postures executed by the subject. The lower variance accounted by the first PCs in this work, with respect to the variance results obtained by Santello et al. in Santello et al. (1998), is related to the fact that in our work synergies were extracted on grasping trials of real objects. Santello et al., instead, extracted synergies by asking subjects to grasp imaginary objects, that is to mimic the hand posture associated to a particular objects by relying only on “grasp memory”. The use of real objects actually reduces the number of PCs to reach a high percentage of variance, due to mechanical constraints associated to the contact with the objects as well as somatosensory feedback (Jarrassé et al. (2014); Santello et al. (2002)). In addition, the reduced set of objects used in our work, which is not identical to the set of objects chosen in Santello et al. (1998), could have generated different synergies in terms of variance and composition.

The computed synergies can be imported in SynGrasp as they are saved into the MATLAB struct which contains the *C* and *S* matrices and the *E*_*val*_ and *E* vectors, to be exploited to perform any desired synergy-based analysis.

Providing the possibility of automatically extracting postural synergies could be useful for developing artificial hands with simplified mechanical structures and controlled by smart control strategies based on synergies. It allows the artificial hands to grasp a variety of objects by actuating a reduced number of DoFs. This approach has been applied for the development of the Hannes Hand (Laffranchi et al. (2020)) and of the PISA IIT SoftHand (Catalano et al. (2014)), two artificial hands which mechanical design is based on the previous computation of human postural synergies. Once designed the artificial hand, it could be very useful to simulate its grasp capabilities before going forward with the fabrication. The SynGrasp toolbox (Malvezzi et al. (2015)) was developed to simulate grasps and analyze the main grasping properties of artificial hands, by also enabling defining compliance at the level of the contact points and joints to investigate the behavior of an artificial hand underactuated on the basis of postural synergies. In particular, the SynGrasp toolbox only incorporates the definition of Santello’s synergies (Santello et al. (1998)), that were extracted by asking five subjects to grasp imaginary objects. Instead, to introduce the possibility of performing grasp analyses on custom-extracted synergies could be really useful, especially given the literature evidence that synergies could be modified by mechanical constraints related to the contact with a real object and/or to somatosensory feedback (Jarrassé et al. (2014); Santello et al. (2002))

The virtual hand model implemented in the tool can be exploited 1) to inspect the posture of the hand in the reach-to-grasp trial for the clinical assessment, and qualitatively evaluate the grasp strategy of the subject; 2) to visualize the movement of the hand according to the activation of postural synergies and 3) to be used in SynGrasp to perform grasp analysis on a custom-designed virtual hand that guarantees a more faithful representation of the hand with respect to the SynGrasp hands.

The tool itself and the analyses presented in this work do not present limitations related to the type of subject analysed. In fact, the tool potentialities do not depend on the subjects to be involved in the analysis. They only relate on the files given as input to the toolbox. In general, the experimental acquisitions could be conducted on any subject, with or without pathologies, as he/she only has to perform simple reach-to-grasp tasks with the hand markerized. The acquisition of marker positions with an optoelectronic motion capture system is a totally non-invasive technique. The type and number of acquisitions to be performed by the subjects can be chosen according to the subject conditions: for example, if the subject presents neuromuscular or musculoskeletal disorders, he/she could have the need to perform fewer repetitions for the clinical assessment and to perform a lower number of reach-to-grasp tasks, by of course choosing the proper set of objects to cover most of the daily life grasps. Moreover, the acquisition procedure could be conducted by introducing pauses between the trials if the subject needs to rest. The illustrative examples provided in the paper were only introduced to show the complete workflow on an experimental acquisition and analysis with the toolbox. However, the age of the subject and their healthy condition did not affect the obtained results in terms of potentialities of the toolbox.

The usability of the toolbox was evaluated in terms of efficiency, effectiveness and satisfaction of the user. As previously introduced in Section 3.3, the time to load data, which includes the loading, pre-processing of data and joint angles computation, is the most time-demanding task of the toolbox. This is due to the number of operations that must be executed to extract joint angles. All the other tasks are executed in a negligible time, ranging from 1 s to visualize plots and hand posture to 4 s to compute indicators and provide results about the clinical assessment. The tool is effective because its structure prevents the possibility of performing errors. Moreover, it is self-contained, as each tab allows executing a different type of task or analysis. The number of navigational steps for each tab is 1, without counting the dialog boxes that allow selecting the files to be loaded. Finally, the perceived usability of the toolbox was very high: subjects assigned a score of 94.2 on average. This means they found the toolbox easy to use and that the toolbox met the requirements. They did not perceive frustration or committed errors in using the tool. This result shows that the proposed MATLAB toolbox could be easily used by non-expert users such as clinical personnel to perform clinical assessment of the hand and postural synergies extraction.

## 5 Conclusion

The open source toolbox presented in this work allows intuitively performing hand clinical assessment by computing meaningful performance indicators that allow evaluating movement efficiency, planning, smoothness, speed and spatial posture. It can also be exploited to evaluate joint range of motion. The tool does not require additional technical knowledge and automatically produces as output a clinical assessment of the patient, therefore it can be easily used by the clinical staff. Moreover, the proposed tool allows extracting postural synergies, to inspect individual motion strategies as well as to define underactuation patterns for artificial biomimetic devices such as hand prostheses and exoskeletons. In addition, it is developed to integrate the SynGrasp toolbox in order to provide the academic and scientific community an integrated environment to extract and exploit postural synergies for any desired analysis.

## Data Availability

The raw data supporting the conclusion of this article will be made available by the authors, without undue reservation.
